# Neural Correlates of Resistance to Gaming Desire Induced by Social Media Content

**DOI:** 10.1111/adb.70085

**Published:** 2025-08-27

**Authors:** Yuka Fujimoto, Junya Fujino, Daisuke Matsuyoshi, Daisuke Jitoku, Nanase Kobayashi, Chenyu Qian, Shoko Okuzumi, Shisei Tei, Takehiro Tamura, Takefumi Ueno, Makiko Yamada, Hidehiko Takahashi

**Affiliations:** ^1^ Department of Psychiatry and Behavioral Sciences, Graduate School of Medical and Dental Sciences Institute of Science Tokyo Tokyo Japan; ^2^ Institute for Quantum Life Science, National Institutes for Quantum Science and Technology Chiba Japan; ^3^ Department of Psychiatry Nara Medical University Nara Japan; ^4^ Medical Institute of Developmental Disabilities Research Showa University Tokyo Japan; ^5^ Joint Research Department of Cyberpsychiatry Institute of New Industry Incubation Institute of Science Tokyo Tokyo Japan; ^6^ Department of Psychiatry, Graduate School of Medicine Kyoto University Kyoto Japan; ^7^ Institute of Applied Brain Sciences Waseda University Saitama Japan; ^8^ School of Human and Social Sciences Tokyo International University Saitama Japan; ^9^ Division of Clinical Research National Hospital Organization, Hizen Psychiatric Medical Center Saga Japan; ^10^ Department of Functional Brain Imaging Institute for Quantum Medical Science, National Institutes for Quantum Science and Technology Chiba Japan; ^11^ Center for Brain Integration Research Institute of Science Tokyo Tokyo Japan

**Keywords:** craving, functional magnetic resonance imaging, gaming addiction, posterior cingulate cortex, precuneus

## Abstract

The rise of gaming‐related content on social media has increased exposure to game‐related stimuli, particularly among young people, which may reinforce gaming urges and create difficulties in controlling gaming behaviour. Therefore, understanding the management of gaming desire triggered by such content is critical. Identifying the neural mechanisms underlying resistance to these urges will be crucial for effective prevention and intervention. However, this issue has yet to be directly explored. The present study investigated the neural correlates of resisting gaming desire elicited by gaming‐related social media videos using functional magnetic resonance imaging (fMRI). Young habitual online gamers participated in an fMRI study in which they viewed video stimuli under three conditions: (1) gaming cue condition: passive viewing of gaming‐related videos; (2) gaming cue resist condition: viewing of gaming‐related videos while actively resisting gaming desire; and (3) neutral cue condition. Gaming cues elicited significantly greater activation than neutral cues in the diverse brain areas including bilateral medial prefrontal cortex, orbitofrontal cortex, anterior cingulate cortex, posterior cingulate cortex (PCC), superior temporal gyrus (STG) and precuneus. Compared to the gaming cue condition, the gaming cue resist condition elicited increased activation in the left PCC and bilateral precuneus. Conversely, significant deactivation was observed in the right STG. These findings offer insights into the neural basis of craving resistance in response to social media‐based gaming cues and may guide the development of targeted interventions for problematic gaming behaviour.

## Graphical abstract

This study investigated the neural correlates of resisting gaming desire elicited by gaming‐related social media videos, using functional magnetic resonance imaging in young habitual online gamers. Compared to the gaming cue condition, the gaming cue resist condition elicited increased activation in the left posterior cingulate cortex and bilateral precuneus. Conversely, significant deactivation was observed in the right superior temporal gyrus. These findings offer insights into the neural basis of craving resistance in response to social media‐based gaming cues.

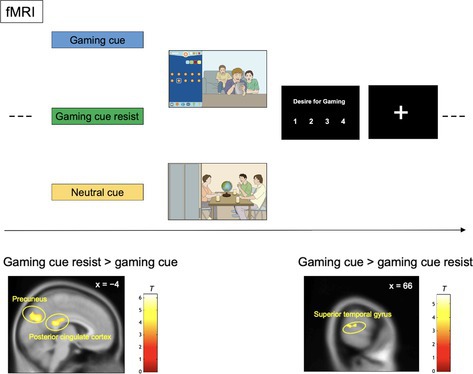

## 1 Introduction

Online games have become immensely popular, not only as entertainment but also as tools for mental, physical and cognitive training [1, 2, 3]. These games offer temporary relief from daily stressors and may be beneficial to psychological well‐being. However, excessive immersion in online gaming can lead to behavioural addiction, potentially displacing meaningful social interactions and causing adverse mental and physical effects [4, 5, 6, 7]. This issue has become a growing concern in the general public and among mental health professionals, particularly for younger populations.

In this era of ubiquitous digital connectivity, online gaming and social media platforms (e.g., TikTok, Instagram and YouTube) have become deeply integrated into everyday life [8, 9, 10]. The increasingly widespread availability of gaming‐related content on such platforms, especially short‐form videos, may potentially evoke gaming urges and foster maladaptive behaviours [10, 11, 12, 13]. Therefore, understanding how individuals manage gaming urges triggered by such content would yield critical insights. Investigating the underlying neural mechanisms behind craving management would facilitate the development of effective prevention and intervention strategies. However, to the best of our knowledge, no studies have directly addressed this issue.

Neuroimaging techniques, such as functional magnetic resonance imaging (fMRI), have shed light on the neural basis of cue‐induced craving in gaming addiction. Previous studies report that, in both healthy gamers and individuals with internet gaming disorder (IGD), gaming cues activate brain regions associated with reward, memory, attention and sensory processing, such as the orbitofrontal cortex (OFC), medial prefrontal cortex (MPFC), anterior cingulate cortex (ACC), posterior cingulate cortex (PCC), hippocampus, superior temporal gyrus (STG) and precuneus [5, 14, 15, 16, 17, 18]. We also previously demonstrated that gaming‐related content on social media elicits similar neural responses, further emphasizing the importance of investigating neural activity in these regions to understand the neurobiological underpinnings of gaming‐related craving [11, 12].

The neural mechanisms involved in controlling cravings in general have also been extensively investigated in addiction research [19, 20, 21, 22]. Brody et al. [19] demonstrated that individuals with nicotine dependence exhibited heightened activation in the dorsal ACC, PCC and precuneus when actively resisting cue‐induced cigarette cravings compared with passive cue exposure. Furthermore, reduced activation was observed in sensory processing regions, such as the occipital cortices, under the same condition. In the context of gaming addiction, recent evidence indicates that individuals with IGD showed dysregulated craving responses, manifesting as altered activation in critical regions for attention processes and cognitive control, including the ACC, PCC, OFC and dorsolateral prefrontal cortex [22]. Building on these findings, we hypothesized that intentional craving resistance following exposure to gaming‐related social media cues would elicit increased activation in the ACC, PCC, precuneus and prefrontal cortices but decreased activation in sensory processing brain regions.

In this study, we employed fMRI to examine the neural correlates of resisting gaming desire induced by social media content in healthy, casual online gamers. Specifically, participants were instructed to view gaming videos under three conditions: (1) gaming cue condition: passive viewing of gaming videos; (2) gaming cue resist condition: viewing of gaming videos while actively resisting gaming urges; and (3) neutral cue condition.

## 2 Materials and Methods

### 2.1 Participants

This study recruited 31 healthy volunteers who engaged in casual online gaming. The sample size was determined based on previous fMRI studies that examined cue reactivity in addiction research [11, 15, 23]. The participants ultimately enrolled were those who played online games regularly for at least 1 h per week and did not meet the Diagnostic and Statistical Manual of Mental Disorders 5th Edition criteria for IGD. After excluding five participants from the analyses because of excessive head motion (> 4 mm), a final total of 26 participants was included in the analysis. In line with previous studies [5, 14], all participants completed the Internet addiction test [24, 25] to evaluate Internet dependence.

The study was approved by the institutional review board of the Institute of Science Tokyo Hospital (R2021‐006) and conformed to the Code of Ethics of the World Medical Association. All participants provided written informed consent after being provided with an explanation of the entire study.

### 2.2 fMRI Task

We modified the task implemented in our previous studies [11, 12]. Eight gaming‐related videos were selected from different social media platforms, featuring scenes of gameplay footage, game launches or tutorials. Each video featured a popular online game in Japan, including shooting, role‐playing, puzzle and sports games. For the control condition, we selected eight neutral videos also sourced from social media but unrelated to gaming, such as content on furniture, hygiene, travel or work. We matched each neutral video to a gaming video in terms of complexity, content, design, luminance, colour, motion and presence of human faces, as described previously [21, 26, 27, 28]. In total, 220 candidate videos were shortlisted and independently rated by three researchers using standardized criteria. Following a group discussion, eight neutral videos that best matched the gaming videos were selected.

**FIGURE 1:**
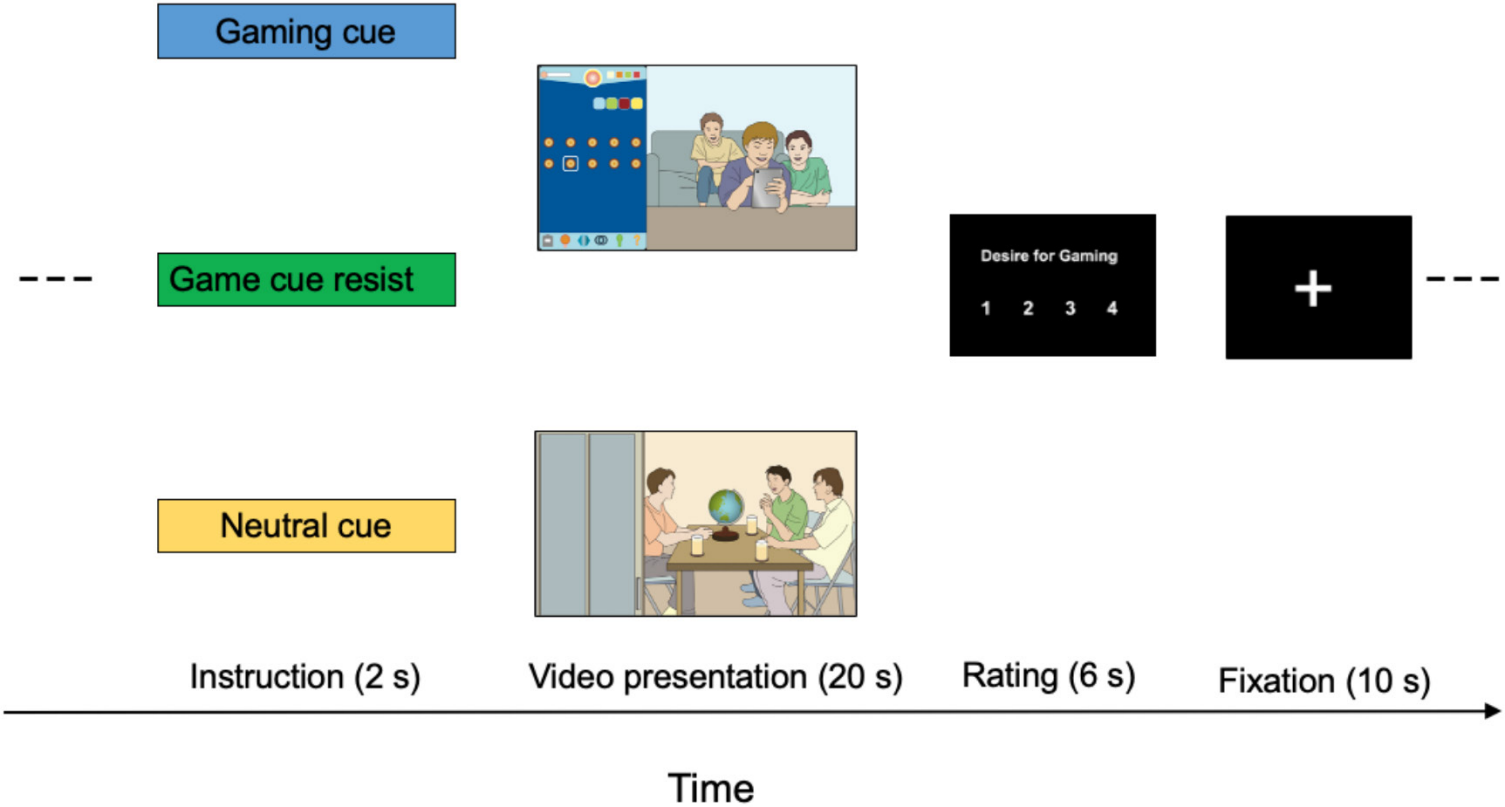
Functional magnetic resonance imaging task. Each video ran for 20 s and was shown in a pseudorandom order. A 2‐s instructional slide was presented before each task block. An illustrative frame from a gaming‐related video used in the task is shown here for reference.

Each video lasted 20 s and was presented in a pseudorandom order (Figure 1). Before each task block, a 2‐s instruction slide was presented. Participants were instructed to view the videos under one of three conditions: (1) gaming cue condition: passive viewing of gaming videos; (2) gaming cue resist condition: viewing of gaming videos while actively resisting gaming urges; and (3) neutral cue condition: passive viewing of neutral videos. Each condition included all eight corresponding videos. After each video, participants were asked to rate their gaming desire on a scale of 1 (*no desire*) to 4 (*extreme desire*), within 6 s. A 10‐s fixation cross was shown between videos. Outside the fMRI scanner, the participants were asked to rate the familiarity of each game they were shown from 1 (*very unfamiliar*) to 9 (*very familiar*). In addition, based on the previous studies [19, 21], they were interviewed about the strategies they used to resist gaming urges.

The experiment was conducted using E‐Prime (Psychology Software Tools Inc., Pittsburgh, PA, USA). Before fMRI, participants completed at least one practice trial on a shorter version of the fMRI task, during which any misunderstandings regarding task completion were clarified.

### 2.3 fMRI Data Acquisition and Preprocessing

All participants underwent MRI scanning using a 3‐T whole‐body scanner (Prisma, Siemens, Erlangen, Germany) equipped with a 20‐channel head/neck coil. SPM12 (Wellcome Trust Center for Neuroimaging, London, UK) in MATLAB (MathWorks, Natick, MA, USA) was used to process images. Further details are presented in Supporting Information.

### 2.4 Data Analysis

#### 2.4.1 Behavioural Data

To examine the effect of resistance, we compared gaming desire in the gaming cue condition with that in the gaming cue resist and neutral cue conditions using paired *t*‐tests based on Dunnett's procedure. Statistical significance was defined as *p* = 0.05 (two‐tailed). All analyses were conducted using SPSS 29 (IBM, Armonk, NY, USA).

#### 2.4.2 fMRI Data

The fMRI data were analysed using a general linear model. For the first‐level analysis, the design matrix incorporated three task‐related regressors of interest: the gaming cue, gaming cue resist and neutral cue conditions. The instructions and rating periods were modeled as covariates of no interest. To minimize motion‐related artefacts, we included six movement parameters (three displacements and three rotations) as regressors of no interest. Subsequently, we characterized the differences in activation between gaming cue and neutral cue conditions, as well as between gaming cue and gaming cue resist conditions. The comparison produced contrast images for each participant, which were then used for second‐level fMRI analyses.

In the second‐level analyses, population‐level inferences were drawn using a random‐effects model. Based on prior literature [14, 16, 17, 18] and in line with our previous studies [11, 12], we focused on specific regions of interest (ROIs), including the MPFC, OFC, middle frontal gyrus (MFG), ACC, PCC, striatum, thalamus, hippocampus, STG and precuneus. Anatomical masks for these ROIs were obtained from the Automated Anatomical Labeling atlas [29, 30]. Significance was determined using family‐wise error (FWE) correction for multiple comparisons, with a cluster‐level threshold of *p* < 0.01 for each ROI (voxel‐level uncorrected *p* < 0.001). Additionally, for exploratory analysis, we reported activations that met a voxel‐level threshold of *p* < 0.01 (FWE corrected) with a minimum cluster extent of 100 contiguous voxels, following whole‐brain correction for multiple comparisons in line with previous studies [11, 12, 31, 32].

To examine the relationship between neural activity and behavioural responses, we performed correlation analyses between the reduction in gaming desire (from gaming cue to gaming cue resist conditions) and the parameter estimates (from gaming cue resist to gaming cue conditions), which were extracted as the first eigenvariate from significant clusters. Correlations were considered statistically significant at *p* < 0.05 (two‐tailed).

## 3 Results

**FIGURE 2:**
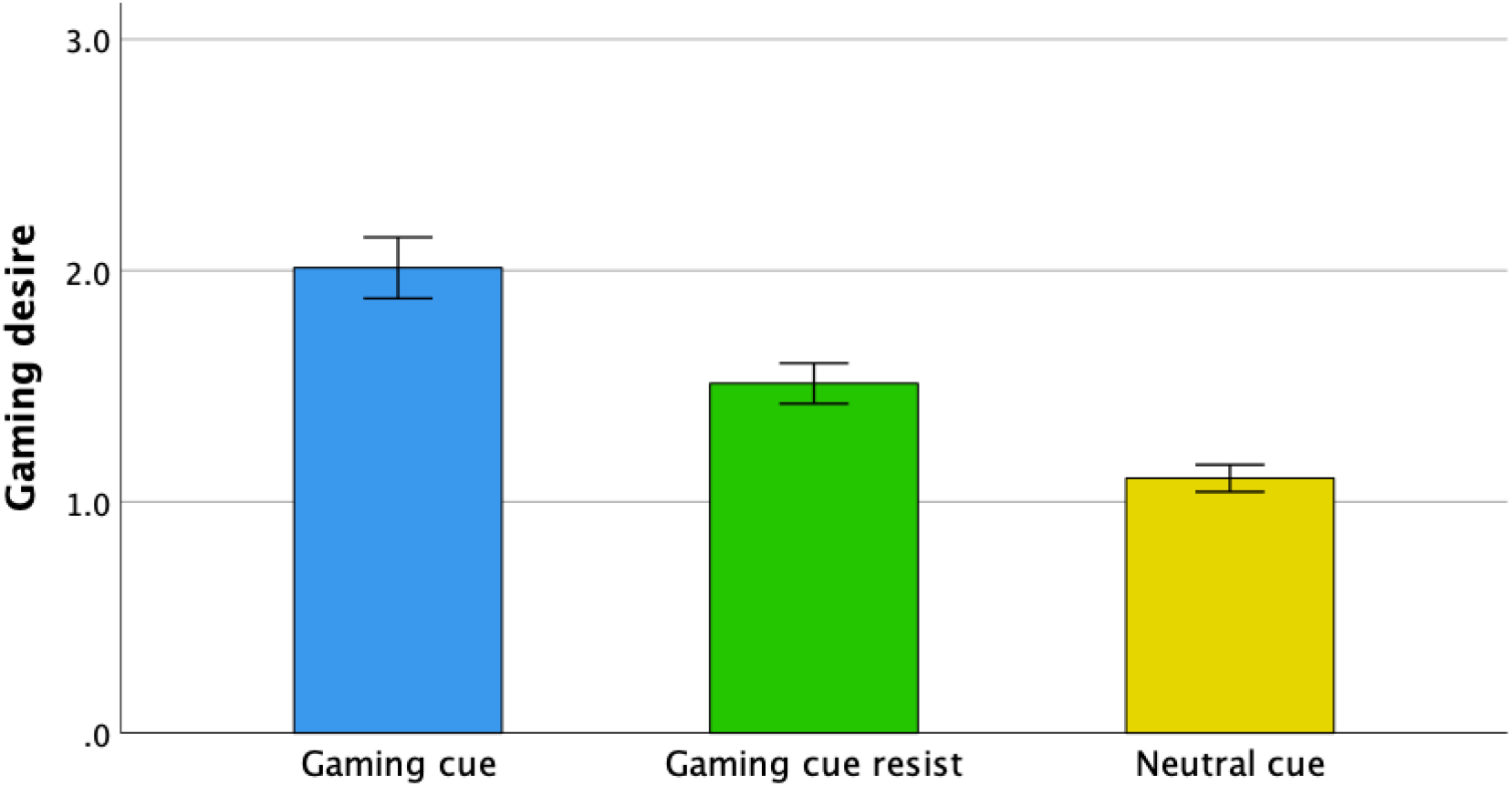
Gaming desire under each condition. The error bars indicate ± standard errors.

Table 1 presents participants' demographic characteristics. Task performance was generally strong, with participants missing an average of only 0.35 ± 0.63 out of 24 trials. A missed trial was defined as one where a participant failed to rate their gaming desire within the allotted time. Gaming desire was significantly higher in the gaming cue condition (2.01 ± 0.67) than in both the gaming cue resist (1.51 ± 0.45) and neutral cue (1.10 ± 0.30) conditions (both *p* < 0.01; Figure 2). No significant correlations were found between gaming desire ratings and familiarity scores in either the gaming cue or gaming cue resist conditions (both *p* > 0.05).

**TABLE 1:** Demographic characteristics of the participants.

	Total (*n* = 26)
Age (years, mean ± SD)	21.6 ± 2.4
Male/female	23/3
Education (years, mean ± SD)	13.2 ± 1.8
Predicted full‐scale IQ (mean ± SD)	107.1 ± 7.2
Internet addiction test (mean ± SD)	35.5 ± 9.4
Weekly gaming time (hours, mean ± SD)	6.1 ± 7.2
Gaming history (years, mean ± SD)	9.9 ± 3.1

Abbreviations: IQ = intelligence quotient; SD = standard deviation.

**FIGURE 3:**
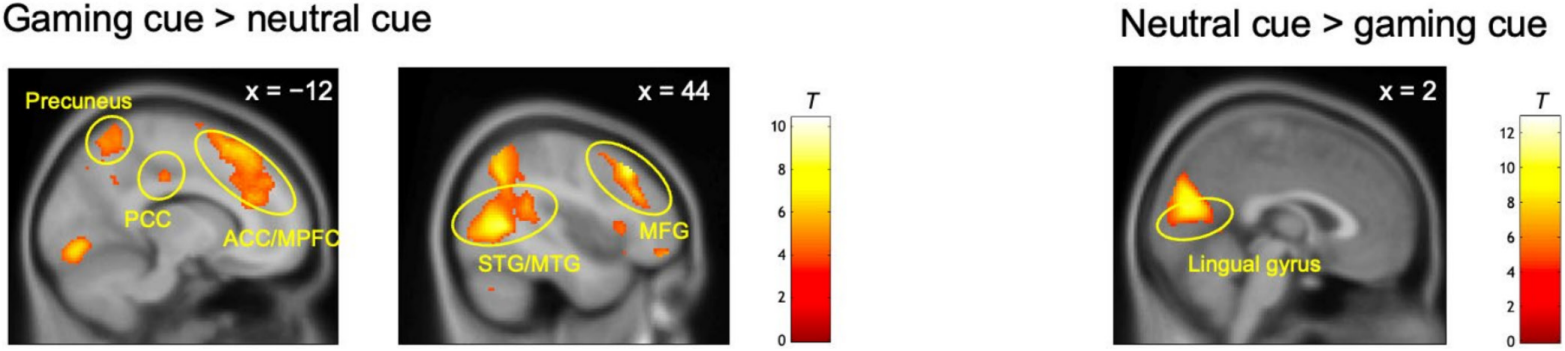
Comparison between gaming and neutral cue conditions. For display purpose, the threshold is set at *p* < 0.001, uncorrected. ACC = anterior cingulate cortex, MFG = middle frontal gyrus, MPFC = medial prefrontal cortex, MTG = middle temporal gyrus, PCC = posterior cingulate cortex, STG = superior temporal gyrus.

The ROI analysis comparing the gaming cue and neutral cue conditions revealed significantly greater activation during gaming cue exposure in the bilateral MPFC, OFC, MFG, ACC, PCC, STG and precuneus (Figure 3 and Table S1). In contrast, a more ventral region of the bilateral precuneus showed significantly greater activation in the neutral cue condition than in the gaming cue condition. Exploratory whole‐brain analysis further revealed that, compared with neutral cue exposure, gaming cue exposure elicited significantly greater activation in the right middle temporal gyrus but led to significant deactivation in the occipital regions, including the bilateral lingual gyrus. Details are described in Table S2.

**FIGURE 4:**
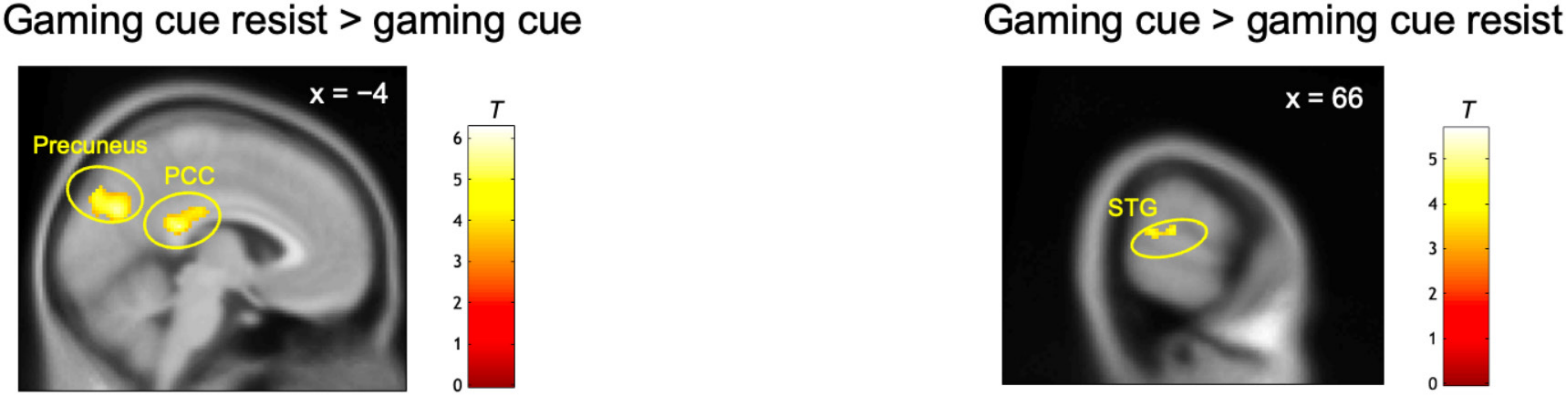
Comparison between the gaming cue resist and gaming cue conditions. For display purpose, the threshold is set at *p* < 0.001, uncorrected. PCC = posterior cingulate cortex, STG = superior temporal gyrus.

ROI analysis comparing the gaming cue resist and gaming cue conditions revealed that the gaming cue resist condition elicited significantly greater activation in the left PCC and bilateral precuneus, coupled with significant deactivation in the right STG (Figure 4 and Table 2). No further significant findings were noted by exploratory whole‐brain analysis.

**TABLE 2:** fMRI results (gaming cue resist condition vs. gaming cue condition [ROI analysis]).

Brain region	Coordinates (mm)				Cluster
	x	y	z	*T*	(voxels)
*Gaming cue resist > gaming cue*					
L posterior cingulate cortex	−8	−40	24	5.48	55
L precuneus	−10	−68	30	6.00	225
R precuneus	4	−72	32	4.78	160
*Gaming cue > gaming cue resist*					
R superior temporal gyrus	58	−36	14	4.34	116

*Note: p* < 0.01, cluster‐level *FWE* corrected (at voxel‐level uncorrected *p* < 0.001). MNI coordinates and *T*‐values were provided for the local voxel maximum of each respective cluster.

Abbreviations: fMRI = functional magnetic resonance imaging, FWE = family‐wise error, L = left, MNI = Montreal Neurological Institute, R = right, ROI = region of interest.

**FIGURE 5:**
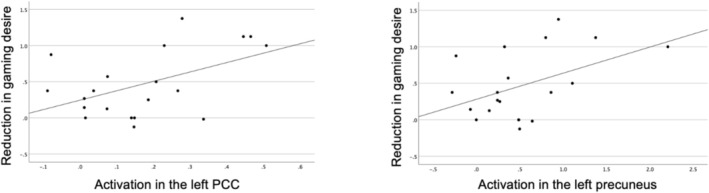
Reduction in gaming desire and activation levels in the left PCC and left precuneus. In the subgroup of participants who reported using strategies related to attentional distraction (*n* = 20), reductions in gaming desire were significantly correlated with activation in the left PCC (*r* = 0.49, *p* = 0.03) and left precuneus (*r* = 0.46, *p* = 0.04). PCC = posterior cingulate cortex.

We also performed correlation analyses between the reductions in gaming desire (from gaming cue to gaming cue resist conditions) and parameter estimates (from gaming cue resist to gaming cue conditions) extracted as the first eigenvariate from significant clusters. No significant correlations were found between the said variables across the entire sample (all, *p* > 0.05). The post‐fMRI interview revealed that 76.9% of participants (*n* = 20) used strategies related to attentional distraction, such as redirecting attention to nongaming‐related thoughts, whereas the other participants either used other strategies or could not verbalize their approach. We therefore performed follow‐up correlation analysis on the participants who reported using strategies related to attentional distraction and found that reductions in gaming desire were significantly correlated with activation in the left PCC (*r* = 0.49, *p* = 0.03) and left precuneus (*r* = 0.46, *p* = 0.04) (Figure 5).

## 4 Discussion

This study examined the neural correlates of resisting gaming urges triggered by social media content in young adults who casually engage in online gaming. Participants reported significantly increased gaming desire in response to gaming‐related social media cues compared with neutral cues during the fMRI task, confirming the significant influence of such content on gaming urges [11, 12, 13]. Notably, gaming desire was significantly lower in the gaming cue resist condition than in the gaming cue condition, indicating that, overall, participants were able to effectively resist gaming urges.

Consistent with our previous findings [11, 12], gaming cues elicited significantly greater activation than neutral cues in the bilateral MPFC, OFC, MFG, ACC, PCC, STG and precuneus. These results underscore the involvement of these regions in processing gaming‐related cues presented on social media among casual online gamers.

Compared with the gaming cue condition, the gaming cue resist condition elicited significantly greater activation in the left PCC and bilateral precuneus. The PCC facilitates directing attention toward internal states, transmitting internally generated information for further evaluation and supporting functional integration [33, 34]. The precuneus is involved in voluntary attention shifts, visuospatial processing and the coordination of spatial behaviour [35, 36]. Notably, both regions are central components of the default mode network, which participates in attentional regulation and cognitive control [37]. The literature has consistently highlighted the involvement of the PCC and precuneus in craving management across both substance and behavioural addictions [20, 22, 38]. Brody et al. [19] reported increased activation in the PCC and precuneus among individuals with nicotine dependence when instructed to resist cravings elicited during cigarette cue exposure. Prashad et al. [39] demonstrated that targeting these brain regions may potentially modulate cravings in cannabis users. Interestingly, in the present study, positive correlations between reductions in gaming desire and increased activation in the left PCC/left precuneus were observed in a subset of participants who employed strategies related to attentional distraction. This finding, which aligns with those of earlier studies [19, 22], suggests that these regions may facilitate attentional shifting processes that help mitigate craving responses.

Conversely, the right STG showed decreased activation during the gaming cue resist condition compared to the gaming cue condition. The STG facilitates the processing of dynamic audiovisual stimuli and social contextual information [40, 41, 42]. Reduced activity in this region may indicate disengagement from the sensory‐rich and socially salient aspects of gaming content as participants actively resisted their gaming urges. This shift in neural engagement may reflect a reallocation of attentional resources from externally oriented perceptual processing toward internally focused regulatory mechanisms. Although this interpretation aligns with the idea of attentional redirection during self‐control [43], it remains speculative and should be directly examined in future studies integrating paradigms that manipulate attentional focus more explicitly.

Contrary to our hypothesis, we did not observe increased activation in the ACC and prefrontal cortices during the gaming cue resist condition. One possible explanation may be the influence of the characteristics of our participant sample. Unlike individuals with clinically diagnosed IGD, our participants were healthy young adults with casual gaming habits. Previous studies have indicated that activation of the ACC and prefrontal regions during craving regulation tasks tends to be more pronounced in clinical populations [21, 22]. In nonclinical individuals, such as those in our sample, the activation of these regions may be more subtle and involve internally guided strategies that do not require robust top‐down control mechanisms detectable at the group level. Future studies should validate the current findings by analysing clinically diagnosed IGD populations to further elucidate the neural mechanisms underlying craving resistance in more severe cases.

From a clinical perspective, the findings of this study offer valuable insights into the management of gaming‐related urges elicited by social media exposure. Given the pervasive presence of gaming content on platforms such as TikTok, Instagram and YouTube, individuals, especially adolescents and young adults, are frequently exposed to stimuli that can trigger cravings and reinforce maladaptive gaming behaviours [8, 9, 10, 13]. Our results indicate that, among healthy casual gamers, actively resisting urges engages neural networks involving the PCC and precuneus, highlighting the potential value of incorporating distraction‐based or internally focused cognitive strategies into psychoeducational programmes for individuals at risk of developing IGD. Furthermore, the integration of real‐world digital media contexts into clinical protocols could increase both the ecological validity and clinical efficacy of craving management strategies.

This study has several limitations. First, as previously noted, the sample was limited to young casual online gamers, excluding individuals with no gaming experience and those clinically diagnosed with IGD. The inclusion of nongamers could have provided valuable insights into determining whether neural responses to gaming‐related stimuli are specific to individuals familiar with gaming culture or whether they also occur in those with little or no gaming exposure. Moreover, neural responses may differ substantially between individuals with subclinical tendencies and those who meet formal diagnostic criteria for IGD [12, 14]. Therefore, the generalization of our findings should be applied with caution. Nonetheless, because IGD often emerges during adolescence and young adulthood, a developmental period marked by heightened exposure to and engagement with online gaming platforms [44, 45], the insights derived from this population may still offer meaningful contributions to the development of effective interventions for IGD. Second, although we closely matched each gaming‐related stimulus with its neutral counterpart, using naturalistic stimuli with real‐world content from social media posed difficulties to achieving perfect matching across multiple parameters. Factors such as the foreground–background ratio and zooming speed remained difficult to fully control despite our efforts to carefully select neutral stimuli. Third, while participants' craving resistance strategies were assessed through post‐experiment interviews, this information was based on self‐reporting and was therefore susceptible to reporting biases. Future studies should compare multiple strategies within the same individuals to uncover the neural mechanisms underlying each approach, as well as to corroborate self‐reported data with objective behavioural measures. Finally, this study utilized a cross‐sectional design, which precludes any causal conclusions. Longitudinal study designs could further clarify the predictive value of the observed neural patterns on long‐term resilience against gaming‐related behavioural dysregulation.

Despite these limitations, the current findings demonstrate that resistance to gaming desire elicited by social media content engages neural networks involving the PCC and precuneus. These results offer valuable insights into the management of gaming‐related urges elicited by digital media exposure.

## Author Contributions

**Yuka Fujimoto:** conceptualization, data curation, formal analysis, investigation, methodology, writing – original draft, writing – review and editing. **Junya Fujino:** conceptualization, data curation, formal analysis, funding acquisition, investigation, methodology, project administration, writing – original draft, writing – review and editing. **Daisuke Matsuyoshi:** data curation, investigation, supervision, writing – review and editing. **Daisuke Jitoku:** conceptualization, data curation, investigation, project administration, writing – review and editing. **Nanase Kobayashi:** conceptualization, data curation, investigation, writing – review and editing. **Chenyu Qian:** data curation, investigation, validation, writing – review and editing. **Shoko Okuzumi:** data curation, investigation, methodology, writing – review and editing. **Shisei Tei:** methodology, supervision, validation, writing – review and editing. **Takehiro Tamura:** conceptualization, investigation, supervision, writing – review and editing. **Takefumi Ueno:** conceptualization, funding acquisition, investigation, methodology, project administration, supervision, writing – review and editing. **Makiko Yamada:** conceptualization, funding acquisition, investigation, methodology, project administration, supervision, writing – review and editing. **Hidehiko Takahashi:** conceptualization, funding acquisition, investigation, methodology, project administration, supervision, writing – review and editing.

## Ethics Statement

The study was approved by the institutional review board of the Institute of Science Tokyo Hospital (R2021‐006) and conformed to the Code of Ethics of the World Medical Association.

## Consent

All participants provided written informed consent after being provided an explanation of the entire study.

## Conflicts of Interest

The authors declare no conflicts of interest.

## Supporting information


**Table S1:** fMRI results (gaming cue condition vs. neutral cue condition [ROI analysis]).
**Table S2:** fMRI results (gaming cue condition vs. neutral cue condition [whole‐brain analysis]).

## Data Availability

The data that support the findings of this study are available from the corresponding author upon reasonable request.
