# Supplementary material for: Neural Correlates of Resistance to Gaming Desire Induced by Social Media Content
Source: Addict Biol. 2025 Aug 27;30(9):e70085. doi: 10.1111/adb.70085 (PMC12381575; doi:10.1111/adb.70085)
Supplement: Supplementary file 1 — Table S1: fMRI results (gaming cue condition vs. neutral cue condition [ROI analysis]). Table S2: fMRI results (gaming cue condition vs. neutral cue condition [whole‐brain analysis]). [file ADB-30-e70085-s001.docx]

**Supplementary Materials**

**Supplementary Methods**

***Participants***

This study recruited 31 healthy volunteers who engaged in casual online gaming. The sample size was determined based on previous fMRI studies that examined cue reactivity in addiction research (Fujimoto et al., 2024; Ko et al., 2013; Lee et al., 2020). The participants ultimately enrolled were those who played online games regularly for at least 1 hour per week and did not meet the Diagnostic and Statistical Manual of Mental Disorders 5th Edition (DSM-5) criteria for IGD. None of the participants had psychiatric disorders—as evaluated by an experienced psychiatrist who used a structured clinical interview for DSM-5—as well as any history of head trauma, severe medical or surgical illness, or substance abuse. After excluding 5 participants from the analyses because of excessive head motion (>4 mm), a final total of 26 participants was included in the analysis. In line with previous studies (Dong et al., 2017, 2021), all participants completed the Internet addiction test (Tateo et al., 2018; Young et al., 1998) to evaluate Internet dependence. Furthermore, the intelligence quotient (IQ) of participants was estimated using the Japanese version of the National Adult Reading Test short form (Matsuoka et al., 2006).

The study was approved by the institutional review board of the Institute of Science Tokyo Hospital (R2021-006)) and conformed to the Code of Ethics of the World Medical Association. All participants provided written informed consent after being provided an explanation of the entire study.

***fMRI data acquisition and preprocessing***

All participants underwent magnetic resonance imaging on a 3-T whole-body scanner equipped with a 20-channel head/neck coil (Prisma, Siemens, Erlangen, Germany). Functional images were captured in a T2*-weighted gradient-echo echo-planar imaging (EPI) sequence. The images were collected using the following parameters: repetition time (TR) = 1000 ms, echo time (TE) = 32 ms, flip angle (FA) = 61°, field of view (FOV) = 231 × 231 mm, and 45 interleaved axial slices at 3.3 mm thick. We did not save the first two volumes to enable signal stabilization. We then acquired the subsequent 962 volumes. Participants individually lay down in a supine position on a scanner bed with a hand-held button-response device. Their vision was corrected, and foam pads were utilized to reduce head motion. They viewed the visual stimuli back-projected onto a screen through a built-in mirror. Structural scans were acquired using T1-weighted three-dimensional magnetization-prepared rapid gradient-echo (3D-MPRAGE) sequences (TR = 2250 ms; TE = 4.5 ms; inversion time = 950 ms; FA = 12°; FOV = 256 × 240 mm; matrix size; 320 × 300; resolution = 0.8 × 0.8 × 0.8 mm^3^; and 224 total sagittal slices).

SPM12 (Wellcome Trust Center for Neuroimaging, London, UK) in MATLAB (MathWorks, Natick, MA, USA) was used to process images. Functional images were corrected for slice-acquisition timing differences and then spatially realigned to adjust for head motion. The T1-weighted anatomical image (3D-MPRAGE) was co-registered with the mean of functional images. Subsequently, the co-registered T1-weighted anatomical image was normalized to a standard T1 template image, as the Montreal Neurological Institute space defines. The parameters from this normalization process were used for each EPI image. Finally, we resampled the anatomically normalized EPI images to a voxel size of 2 mm × 2 mm × 2 mm and spatially smoothed them using a Gaussian kernel with a full width at a half-maximum of 8 mm in the x, y, and z axes.

**Supplementary References**

Dong, G., Wang, L., Du, X., & Potenza, M. N. (2017). Gaming increases craving to gaming-related stimuli in individuals with Internet gaming disorder. Biological Psychiatry: Cognitive Neuroscience and Neuroimaging, 2(5), 404-412.

Dong, G. H., Wang, M., Zheng, H., Wang, Z., Du, X., & Potenza, M. N. (2021). Disrupted prefrontal regulation of striatum-related craving in Internet gaming disorder revealed by dynamic causal modeling: Results from a cue-reactivity task. Psychological medicine, 51(9), 1549-1561.

Duvernoy, H.M., 1991. The Human Brain. Surface, Three-dimensional Sectional Anatomy and MRI. Springer-Verlag, Wien.

Fujimoto, Y., Fujino, J., Matsuyoshi, D., Jitoku, D., Kobayashi, N., Qian, C., ... & Takahashi, H. (2024). Neural responses to gaming content on social media in young adults. Behavioural Brain Research, 467, 115004.

Ko, C. H., Liu, G. C., Yen, J. Y., Yen, C. F., Chen, C. S., & Lin, W. C. (2013). The brain activations for both cue-induced gaming urge and smoking craving among subjects comorbid with Internet gaming addiction and nicotine dependence. Journal of psychiaric research, 47(4), 486-493.

Lee, J., Lee, D., Namkoong, K., & Jung, Y. C. (2020). Aberrant posterior superior temporal sulcus functional connectivity and executive dysfunction in adolescents with internet gaming disorder. Journal of Behavioral Addictions, 9(3), 589-597.

Matsuoka, K., Uno, M., Kasai, K., Koyama, K., & Kim, Y. (2006). Estimation of premorbid IQ in individuals with Alzheimer’s disease using Japanese ideographic script (Kanji) compound words: Japanese version of National Adult Reading Test. Psychiatry and clinical neurosciences, 60(3), 332-339.

Talairach, J., Tournoux, P., 1988. Co-planar Stereotaxic Atlas of the Human Brain: 3-Dimensional Proportional System—An Approach to Cerebral Imaging. Thieme, New York.

Tateno, M., Teo, A. R., Shiraishi, M., Tayama, M., Kawanishi, C., & Kato, T. A. (2018). Prevalence rate of Internet addiction among Japanese college students: Two cross‐sectional studies and reconsideration of cut‐off points of Young's Internet Addiction Test in Japan. Psychiatry and clinical neurosciences, 72(9), 723-730.

Tzourio-Mazoyer, N., Landeau, B., Papathanassiou, D., Crivello, F., Etard, O., Delcroix, N., Mazoyer, B., Joliot, M., 2002. Automated anatomical labeling of activations in SPM using a macroscopic anatomical parcellation of the MNI MRI single-subject brain. Neuroimage 15(1), 273-289.

Young, K. S. (1998). Caught in the net: How to recognize the signs of internet addiction- and a winning strategy for recovery. New York: Wiley.

**Table S1. fMRI results (gaming cue condition vs. neutral cue condition [ROI analysis])**

| Brain Region | Coordinates (mm) | | | *T* | Cluster |
| --- | --- | --- | --- | --- | --- |
|  | x | y | z |  | (voxels) |
| *Gaming cue > neutral cue* |  |  |  |  |  |
| L anterior cingulate cortex | −12 | 36 | 24 | 5.40 | 277 |
| L middle frontal gyrus | −36 | 20 | 50 | 6.63 | 448 |
| L medial prefrontal cortex | −4 | 40 | 46 | 6.39 | 1199 |
| L orbitofrontal cortex | −36 | 46 | −2 | 5.23 | 216 |
| L posterior cingulate cortex | 0 | −44 | 28 | 6.45 | 304 |
| L superior temporal gyrus | −56 | −16 | 2 | 10.19 | 1047 |
| L precuneus | −6 | −56 | 64 | 5.20 | 362 |
| R anterior cingulate cortex | 6 | 36 | 28 | 5.35 | 412 |
| R middle frontal gyrus | 42 | 24 | 42 | 8.73 | 849 |
| R medial prefrontal cortex | 12 | 34 | 52 | 8.14 | 711 |
| R orbitofrontal cortex | 36 | 42 | −16 | 8.04 | 350 |
| R posterior cingulate cortex | 6 | −48 | 24 | 7.14 | 185 |
| R superior temporal gyrus | 64 | −6 | −2 | 9.61 | 1605 |
| R precuneus | 4 | −52 | 24 | 6.58 | 419 |
|  |  |  |  |  |  |
| *Neutral cue > gaming cue* |  |  |  |  |  |
| L precuneus | −8 | −58 | 8 | 7.29 | 159 |
| R precuneus | 16 | −58 | 14 | 8.48 | 196 |

*p* < 0.01, cluster-level *FWE* corrected (at voxel-level uncorrected *p* < 0.001)

MNI coordinates and *T*-values were provided for the local voxel maximum of each respective cluster.

The largest cluster was reported in the case with more than one cluster in the ROI.

Abbreviations: FWE = family-wise error, L = left, MNI = Montreal Neurological Institute, R = right, ROI = region of interest

**Table S2. fMRI results (gaming cue condition vs. neutral cue condition [whole brain analysis])**

| Brain Region | Coordinates (mm) | | | *T* | Cluster |
| --- | --- | --- | --- | --- | --- |
|  | x | y | z |  | (voxels) |
| *Gaming cue > neutral cue* |  |  |  |  |  |
| L superior temporal gyrus | −56 | −16 | 2 | 10.19 | 274 |
| R superior temporal gyrus | 64 | −6 | −2 | 9.61 | 115 |
|  | 60 | −12 | 2 | 8.45 |  |
| R middle temporal gyrus | 44 | −62 | 6 | 8.09 | 163 |
|  |  |  |  |  |  |
| *Neutral cue > gaming cue* |  |  |  |  |  |
| L lingual gyrus | −12 | −56 | 0 | 11.95 | 1606 |
| R calcarine sulcus | 16 | −66 | 10 | 10.54 |  |
| R lingual gyrus | 12 | −54 | 0 | 9.97 |  |

*p* < 0.01 (FWE corrected) and *k* = 100 voxels

Three local maxima of >8.0 mm apart are reported.

We interpreted the anatomical location of the clusters by consulting the Talairach Daemon database (http://www.talairach.org), the Anatomic Automatic Labeling toolbox, (Tzourio-Mazoyer et al., 2002), and neuroanatomy atlas textbooks (Duvernoy, 1991; Talairach and Tournoux, 1988).

Abbreviations: FWE = family-wise error, L = left, MNI = Montreal Neurological Institute, R = right, ROIs = regions of interest
